# Predictors of Participation of Sophomore Medical Students in a Health-Promoting Intervention: An Observational Study

**DOI:** 10.1371/journal.pone.0168104

**Published:** 2016-12-12

**Authors:** Thomas Kötter, Johanna Ritter, Alexander Katalinic, Edgar Voltmer

**Affiliations:** 1 Institute of Social Medicine and Epidemiology, University of Lübeck, Lübeck, Germany; 2 Department of Health Sciences, Friedensau Adventist University, Möckern, Germany; Wuhan University, CHINA

## Abstract

Medical students and doctors have to be particularly stress-resilient, as both medical education and practice are considered very stressful. Specific stressors can lead to increased risks of developing, for example, depression, anxiety and burnout. Relaxation techniques have proven to be effective for the prevention of these outcomes in student populations. However, only a very few medical students practice relaxation techniques regularly early on in their studies. Furthermore, it is unclear which students make use of stress-management offers and hence whether vulnerable students are generally reachable. Therefore, the aim of our study was to explore predictors of participating in a voluntary stress management course for sophomore medical students. One cohort of freshmen at a German medical school was surveyed at the end of the freshman year [t1] and at the end of the sophomore year [t2]. In addition to sociodemographic information, we captured perceived study stress, self-rated general health and mental health and dimensions of study-related behaviour and experience as potential predictors of participation at t1. During the sophomore year, we offered the participants a progressive muscle relaxation (PMR) beginners’ course. At t2, we registered participation status. We used binary logistic regression analyses in order to assess correlations between potential predictors and participation. About one third of the whole class took part in the course. The main reason for non-participation was “no time”. Being female and higher levels of anxiety were the strongest predictors of course participation. *Career ambition* (the higher, the less likely to participate) and *emotional distancing* (the higher, the more likely to participate) were further significant predictors. Future interventions should be attractive to both male *and* female medical students. Ideally, for every hour of stress management teaching, the curriculum should be cut by at least the same amount of time.

## Introduction

The medical degree is often described as a particularly demanding programme. In addition to non-specific academic and all-day life stress, specific medical school stressors can serve as negative input to the so-called medical students’ coping reserve [1]. In combination with certain personality and temperament factors specific to medical students and / or physicians, such as obsessionality and compulsiveness [2], these stressors can lead to an increased risk of developing depression, anxiety, burnout and other stress-related illnesses [3–7]. In order to be able to heal the sick and stay healthy themselves, medical students and doctors should be–or become–particularly stress-resilient [1,7].

Amongst other stress-management interventions, relaxation techniques have proven to be especially effective in (medical) student populations [8,9]. Learning to practice a relaxation technique was identified by students as a potentially useful health-promoting intervention [10]. Unfortunately, only a very few medical students practice relaxation techniques regularly at the beginning of their studies [11]. Reasons for non-use might be a lack of knowledge of appropriate techniques and the potential benefits and a lack of time.

In Germany, progressive muscle relaxation (PMR) and autogenic training are among the most common relaxation techniques. Both can be performed independently without further aids or equipment after only a short period of instruction. As an effort to introduce health-promoting interventions to the medical curriculum as a positive input to the medical students’ coping reserve, learning and practicing one of these techniques could be a promising approach at a reasonable cost-benefit ratio [8,9]. In order to reach the students most likely to benefit from this kind of intervention, a first step should be to learn who might participate in a voluntary PMR course and who, most likely, does not. The literature investigating this question in the context of medical education is scarce. To our knowledge, to date, there exists only one article by van Dijk et al. exploring participation of medical students in a stress management intervention [12].

The aim of our study was, therefore, to explore individual predictors of participating in a voluntary course in progressive muscle relaxation for sophomore medical students.

## Materials and Methods

### Study design and setting

The data for the above mentioned objective were gathered as part of an ongoing prospective, longitudinal study (Lübeck University Students Trial) at the University of Lübeck, Germany, a public university with a focus on medicine and life sciences. For a detailed description of the study design, especially the validated instruments used, see Kötter et al., 2014 [11].

### Study size

The study size was pre-defined by the number of freshmen and sophomore students at the University of Lübeck. We asked one complete class (n = 189 medical students) to participate.

### Participants and surveys

We invited all medical freshmen from the 2011 cohort at the University of Lübeck. The t1 survey was taken in June 2012, at the end of the freshman year. The intervention took place at the beginning of the summer semester 2013. The follow-up survey (t2) was taken in June 2013, at the end of the sophomore year. Both surveys were web-based. There were no exclusion criteria.

### Intervention

We offered the participants a PMR beginners course, conducted by an experienced PMR instructor. The course contained two modules, each 45 minutes long. The first module was an introduction to PMR, followed by a refresher module several weeks later. We defined complete participation as having attended at least one introduction module *and* one refresher module. We offered five introduction modules and four refresher modules in order to enable all students to attend both modules. Participating in all nine modules was possible.

The course took place in a seminar room on campus and in a time slot with no other competing mandatory classes.

### Measures

At the end of the freshman year (t1), in addition to the information gathered about participant age and gender, we captured different aspects of psychological health, including perceived study stress, self-rated general health and mental health and dimensions of study-related behaviour and experience.

We measured perceived medical school stress using the perceived medical school stress scale in the German language (PMSS-D) [13]. This instrument was originally developed by Vitaliano et al. in 1984 [14]. It has been translated into different languages and cultures and comprises 13 items in the German version. Each item can be answered on a five-point Likert scale (1 = I strongly disagree; 5 = I strongly agree). PMSS results have demonstrated good test-retest-reliability [13], have been linked to physical and mental well-being [13] and have predictive validity for mental health problems in medical professionals four years after graduation [15].

Self-rated general health was measured by a single item (“How would you describe your health in general?), to be answered on a five-point Likert scale from “very good” to “very poor” [16]. Single item self-rated health has been found to be a predictor for several health outcomes in previous studies, including mortality [17]. The item showed good reliability, as well as strong concurrent and discriminant validity in earlier research [18].

In order to measure mental health, we used the Hospital Anxiety and Depression Scale (HADS) [19]. The HADS was initially developed for clinical populations, its psychometric properties have been thoroughly reviewed and it is considered to be a reliable and valid instrument for the assessment of anxiety and depression [20]. It has been widely used among students in general and medical students in particular [11,21,22]. The HADS comprises fourteen items for two sub-scales. Each of the two sub-scales relating to anxiety and depression, respectively, consists of seven items which obtain responses on a four-point Likert scale, ranging from 0 = mostly to 3 = not at all. Possible sub-scale scores range from 0 to 21. We used the German version (HADS-D), published by Herrmann-Lingen et al. in 1995 and available in the 3rd edition [23].

The short form of the German questionnaire AVEM (“Arbeitsbezogene Verhaltens- und Erlebensmuster”, Work study-related behaviour and experience; 44 items; [24]) covers behaviour-related health risks or resources in 11 dimensions, which can be assigned to three major domains: (1) professional commitment; (2) resistance towards stress; and (3) emotional well-being (in the context of work). These dimensions have been shown to predict health outcomes in previous studies [24]. The AVEM has been used in numerous studies with (medical) students [5,25,26].

At the end of the sophomore year (t2), we registered participation status, reasons for non-participation and opinions on stress management interventions in the medical curriculum in general.

### Preventing selection bias

To reduce potential drop-out rates, participants received a book voucher to the value of 5 Euro per attended PMR-session and per completed questionnaire (t1 and t2).

### Data management

From both surveys, data were exported directly into an SPSS format file. We substituted missing values following the rules provided in the handbooks for the instruments, i.e. through interpolation, where tolerable [23,24]. We then excluded incomplete data-sets. After a plausibility check, cases from the t1 and t2 surveys were matched using a self-generated pseudonym.

### Statistics

Data analyses were conducted with IBM SPSS statistics, version 20. After a plausibility check, data from the t1 questionnaires were merged and summarised using descriptive statistics to provide a comprehensive profile of this cohort. We used unpaired t-tests to compare means of continuous variables (see section Measures above) between participants and non-participants and report results as means±standard deviation. For gender, data were analysed using a chi-square-test and the result reported as a percentage. All statistical tests were performed two-tailed with an alpha of .05. Due to the exploratory character of this study, we waived an adjustment for multiple testing [27].

We used binary logistic regression analyses in order to assess correlations between potential predictors and intervention participation. In order to remove variables in the logistic regression analyses, we employed stepwise backwards elimination with p>.05 as the level for removing effects, while keeping age and gender as important sociodemographic variables in order to control for them.

### Ethical issues

The study was approved by the Ethics Committee of the University of Lübeck (File reference 11–010). All participants provided their written informed consent to participate in this study. The ethics committee approved this consent procedure. This report was written in consideration of the STROBE-Statement [28].

## Results

### Study participants

**Fig 1:**
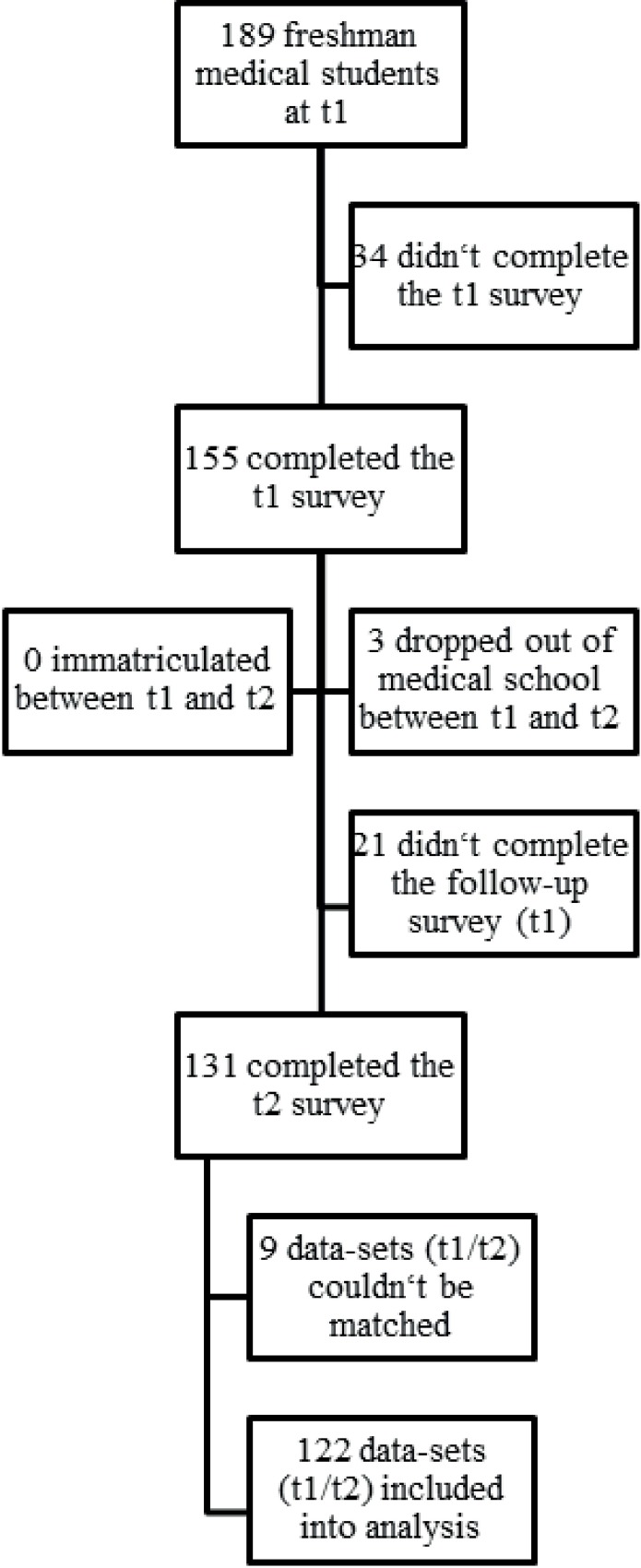
Participant flow.

After exclusion of incomplete data-sets, 122 t2-cases could be matched to t1-cases (93% of the 131 t2-respondents and 66% of all students matriculated at t2). The longitudinal cohort showed a slightly higher proportion of female students (69%) when compared to the cross-sectional samples at t1 (67%), t2 (67%) and all enrolled students at both points in time (64%). The mean age of the longitudinal cohort at t1 (20.6 years) was comparable to that of the cross-sectional sample (20.9) and all enrolled students (21.2) at t1. For an overview of the participant flow, see Fig 1. Baseline characteristics for the longitudinal sample are displayed in Table 1. When comparing male and female respondents, we saw higher levels of *depression* and *balance and mental stability* in male students and higher levels of *striving for perfection* in female students.

**Table 1:** Baseline (t1) characteristics.

	Overall	Female	Male
***n (%)***	122	84 (68.9)	38 (31.1)
***Response (%)***	65.6	71.2	55.9
***M* Age *(SD)* in years t1**	21.3 (2.9)	21.4 (3.0)	21.2 (2.5)
*Overall health*			
***M* General Health *(SD)***	2.3 (0.8)	2.3 (0.8)	2.2 (0.7)
*Mental health*			
***M* Anxiety *(SD)***	5.2 (3.0)	5.3 (3.0)	5.2 (3.0)
***M* Depression *(SD)***	2.9 (2.1)	2.6 (2.0)	3.5 (2.2)
*Perceived Medical School Stress*			
***M* Perceived Medical School Stress *(SD)***	31.1 (6.4)	31.0 (6.2)	31.2 (6.9)
*Dimensions of study-related behavior and experience*			
***M* Subjective significance of work *(SD)***	11.8 (2.8)	11.8 (2.8)	11.6 (2.7)
***M* Career ambition *(SD)***	13.6 (2.5)	13.4 (2.4)	13.8 (2.6)
***M* Tendency to exert *(SD)***	11.8 (3.0)	11.9 (3.1)	11.5 (3.0)
***M* Striving for perfection *(SD)***	13.4 (2.9)	13.7 (3.0)	12.7 (2.8)
***M* Emotional distancing *(SD)***	12.3 (2.7)	12.2 (2.6)	12.6 (2.7)
***M* Resignation tendencies *(SD)***	11.9 (2.8)	12.1 (2.9)	11.6 (2.8)
***M* Offensive coping with problems *(SD)***	13.4 (2.8)	13.2 (2.7)	13.8 (2.9)
***M* Balance and mental stability *(SD)***	13.3 (3.1)	12.8 (3.0)	14.4 (3.0)
***M* Satisfaction with work *(SD)***	16.7 (2.5)	16.7 (2.5)	16.6 (2.4)
***M* Satisfaction with life *(SD)***	16.3 (2.5)	16.4 (2.5)	16.1 (2.5)
***M* Experience of social support *(SD)***	17.2 (2.6)	17.3 (2.5)	17.1 (2.9)

### Intervention participants

Of the 122 students surveyed in this study, 75 (62%) took part in an introductory module on PMR. For a full participation as defined above, participation was required in both the introductory *and* the refresher module, which was the case for about one third of all respondents (n = 45; 37%). When asked for the reason for non-participation, the most frequent answer was “no time” (n = 58, 69%). Further reasons were a lack of interest (n = 11, 14%), having missed the introduction module (n = 4, 5%) or the fact that PMR was already known (n = 5, 7%).

Potential predictors of participation in the intervention with means / percentages of participants and non-participants are displayed in Table 2. Participants were statistically significantly more often female when compared to non-participants (84% vs. 50%; p < .05). Intervention participants were the same age, rated their general health equally and showed the same level of depression when compared to non-participants. Of note, participants showed a relevant difference in their mean score for anxiety (6.0) when compared to non-participants (4.8). However, this difference was not statistically significant (p = .09). There were no further bivariate statistically significant differences between participants and non-participants regarding potential predictors of participation (perceived medical school stress, dimensions of study-related behaviour and experience).

**Table 2:** Bivariate differences between intervention participants and non-participants.

	Participants	Non-participants	*t(df)*	*p*
***n (%)***	45 (36.9)	77 (63.1)	n/a	n/a
***n* female *(%)***	38 (84.4)	46 (49.7)	8.35^1^	< .05*
***M* Age *(SD)* in years [t1]**	21.4 (2.9)	21.3 (2.8)	-0.27(119)	.79
*Overall health*				
***M* General Health *(SD)***	2.2 (0.7)	2.3 (0.8)	0.34(119)	.74
*Mental health*				
***M* Anxiety *(SD)***	6.0 (3.7)	4.8 (2.5)	-1.88(92)	.09
***M* Depression *(SD)***	2.9 (2.3)	2.9 (2.0)	0.07(113)	.95
*Perceived Medical School Stress*				
***M* Perceived Medical School Stress *(SD)***	31.3 (7.6)	30.9 (5.7)	-0.29(116)	.77
*Dimensions of study-related behavior and experience*				
***M* Subjective significance of work *(SD)***	11.6 (2.9)	12.1 (2.7)	0.94(119)	.35
***M* Career ambition *(SD)***	13.1 (2.8)	13.8 (2.4)	1.51(119)	.14
***M* Tendency to exert *(SD)***	12.0 (2.9)	11.8 (3.1)	0.29(119)	.77
***M* Striving for perfection *(SD)***	13.2 (3.2)	13.5 (3.0)	-0.46(119)	.64
***M* Emotional distancing *(SD)***	12.4 (2.7)	12.1 (2.5)	-0.50(119)	.62
***M* Resignation tendencies *(SD)***	12.4 (2.7)	11.8 (2.5)	-0.90(118)	.37
***M* Offensive coping with problems *(SD)***	13.2 (2.6)	13.7 (2.9)	0.51(118)	.61
***M* Balance and mental stability *(SD)***	13.2 (2.8)	13.2 (3.3)	-0.37(119)	.71
***M* Satisfaction with work *(SD)***	16.8 (2.7)	16.8 (2.5)	0.11(119)	.91
***M* Satisfaction with life *(SD)***	16.1 (2.6)	16.4 (2.3)	-0.56(119)	.58
***M* Experience of social support *(SD)***	17.1 (3.1)	17.4 (2.3)	-0.20(118)	.85

(1) chi-square

(*) statistically significant difference between groups

### Predictors of participation

Logistic regression revealed that being female, higher levels of *anxiety* and *emotional distancing*, as well as a lower level of *career ambition* were statistically significant predictors of participation in the intervention. Age did not prove a statistically significant predictor (Table 3).

**Table 3:** Predictors of participation.

Predictor	Range	Odds ratio	95% CI^1^	p
**Age**	18–41	0.89	0.71–1.06	.17
**Gender**	0 male1 female	4.01	1.26–12.76	.02
**HADS Anxiety**	0–21	1.56	1.18–2.06	< .01
**Emotional distancing**	0–16	1.31	1.00–1.71	.05
**Career ambition**	0–16	0.76	0.60–0.97	.03

Nagelkerkes R^2^ = .34

(1) confidence interval

## Discussion

In our prospective, longitudinal study exploring predictors of participation in a voluntary health-promoting intervention for sophomore medical students, we found that being female and higher levels of anxiety had the strongest influence on the likelihood of students attending such a course.

### Participation

Nearly two thirds of the students attended a voluntary introductory module on PMR. However, only half of them also attended the refresher module and can thus be called intervention participants as defined above. This participation rate still lies within that of other studies on stress management intervention in medical education [29–32]. A similar dropout rate during a voluntary wellness programme for medical students has been described by McGrady et al. [33]. Given the heavy course load in the sophomore year, the initial attendance of nearly two thirds might be an indicator of the students’ perceived need of stress management skills. This is further underlined by the fact that 83% of the study participants stated that they regard stress management courses in medical education as “useful”. This resembles the finding of Aster-Schenck et al., that 85% of medical students from all semesters wish for stress management courses [26]. Most of the dropouts gave “no time” as the reason for not attending the refresher course–another hint at the vicious circle of high workload and no time to learn how to deal with it. “No time” being the main reason for non-participation is also in line with the findings of the above mentioned study by Dijk et al. exploring participation in a stress management course for medical students [12].

### Predictors of participation

Being female proved to be the strongest predictor for participation in our PMR course in the logistic regression analysis. This finding is consistent with results from studies of the utilisation of health-promoting interventions in the general population [34] as well as results of a similar study in the context of medical education [9]. It could be an indicator that a PMR course as designed for this study is not attractive enough for male medical students. In part, it might reflect the generally lower willingness of men to take preventive measures for their health [35]. The second strong predictor for participation in the PMR course was the level of anxiety (the higher, the more likely to participate). Students with a high level of anxiety could benefit most from such an intervention, as the efficacy of PMR for the improvement of anxiety is well-described [36]. A number of studies described a higher vulnerability in female medical students regarding anxiety when compared to male [37–39]. This is of note since in many western societies the number of female medical students is larger than the number of male medical students [40]. However, in our data we did not find such a gender difference.

In addition, we found that two dimensions of study-related behaviour and experience, namely *career ambition* (the higher, the less likely to participate) and *emotional distancing* (the higher, the more likely to participate), were statistically significant predictors. It seems that students with a healthier coping style were more likely to attend. Students with higher levels of *career ambition* and an impaired ability for *emotional distancing* from their study subject might not feel able to give time to a stress management course. This could prove another vicious circle, as these individuals are at a higher risk of burnout in the course of their further education [25].

### Strengths and limitations

Our study is one of the first exploring predictors of participation in a stress management course for medical students. The longitudinal design of our study has the advantage of being able to identify “real” predictors in the sense that the potentially participation-predicting attributes were measured significantly ahead of the intervention. We collected data using a web-survey, which may have reduced bias due to social desirability.

Because the study was single-centred, the generalisability of our results may be limited. In addition, compared to the whole class, our longitudinal cohort is relatively small (66%), bearing the risk of selection bias. However, by comparing our cohort to the whole class in terms of gender and age, we could rule out major problems regarding representativeness.

Due to the relatively small sample size, our study had a rather exploratory character. The likelihood of type 1 error is increased due to waiving an adjustment for multiple testing. The findings, even if consistent with earlier research, therefore need to be interpreted with caution and have to be confirmed in larger studies.

### Implications for research and practice

The high initial participation rate on the one hand, and the high dropout rate during the intervention on the other hand, highlight the need to design stress management courses for medical students *and* to make room for them in the curriculum at the same time. Such interventions should either be more attractive for male medical students or there should be different types of stress management courses for different interest groups. The needs and wishes of male medical students could be further explored through qualitative studies. The next step for the evaluation of stress management courses for medical students should be high-quality, adequate-sized randomised controlled trials to test their (long-term) efficacy for preventing the deterioration of medical students’ health.

## Conclusions

In conclusion, our explorative study on the participation of sophomore medical students in a stress management course showed few strong predictors of participation. With a short PMR course, we reached more women and students with higher levels of anxiety. Future interventions should be attractive to both male *and* female medical students. Ideally, in order to keep participation rates high, for every hour of stress management teaching, the curriculum must be cut by at least the same amount of time.
